# Microscopy of the Teeth

**Published:** 1868-02

**Authors:** S. P. Cutler


### DENTAL REGISTER.

Vol. XXII.] FEBRUARY, 1868. [No. 2.

### Original Communications.

### MICROSCOPY OF THE TEETH—CONTINUED.

BY S. P. CUTLER, M. D., A. E. G., D. D. S.

Professor of Chemistry and Microscopy in the Ohio Dental College, Cincinnati. Formerly Professor of Chemistry and Natural Sciences in the Botanic Medical College, Memphis, Tenn.

I shall devote a few pages to fang development, especially in the permanent molars, or rather the closing section of that process, which has not received due and special attention as yet.

I shall commence with the six year molars. Preparations from the French.

#### FIRST SERIES.

#### CASE 1—6 YEARS OLD.

In this specimen the four first molars are in the lower jaw just through the alveolæ, and on a level with the surface of the bone; the fangs extended just below the bifurcations with very thin sharp edges, enveloped by its crypt, forming a closed bony envelope, except at the cutting surface where there is an opening, as is usually the case with all teeth.

The superior anterior molars are elevated nearly one-fourth above the alveolæ, the fangs a little longer than lower, their crypts in contact with floor of the antrum. The same funnel shaped openings in fangs as in the lower.

The lower central incisors advancing at their fangs which are about half grown. The laterals about one-half as much fang as the centrals; none of the balance have advanced any beyond their necks.

### DECIDUOUS ALL INTACT.

### CASE 2—7 YEARS OLD.

In this case the four centrals are full grown, the laterals below nearly so; the upper laterals not erupted though advancing.

The fangs of the lower are nearly perfect, the apex of the upper not complete; slightly funnel shaped. The enamel grooved and spotted extensively.

The lower molars are erupted, the fangs nearly grown, open and funnel shaped at apex, sharp and thin, nearly resting on the nerve. The upper molars advancing.

### CASE 3—11 YEARS OLD.

In this case the four lower incisors are full grown, also the superior centrals, and four anterior molars. The upper laterals erupted and about three-fourths grown. The others following their usual course, only backward. The lower incisors have each two grooves in their cutting edges, the uppers only one groove in each. In this case the fangs are about two-thirds grown, funnel shaped and very thin walls, and large canal, points square across, and full width of the fang.

In this case all the incisors have funnel shaped openings and their edges thin, showing a non-completion at apex, less so than either of the others spoken of.

### CASE 4—9 YEARS OLD.

In this case six of the first molars have not yet left the jaws, but are riding on their permanent contemporaries, ready to drop out. The four incisors above and below are full grown, also the lower canines and two bicuspids nearly.

The four first permanent molars are full grown; the four second are advancing, and have made their appearance above the jaw; the fangs about half grown.

The upper incisors are nearly closed at apex of fang; three of the molars are closed. One of the first inferior is open and funnel shaped at apex through full length. The lower cuspids are grown, but open and funnel shaped apex.

The erupted and non-erupted bicuspids are nearly grown, but very open and funnel shaped. The upper canines have nearly grown fangs, but have not yet advanced any towards eruption.

In this case the cutting edges of incisors are slightly notched, otherwise healthy.

All the above cases indicate bad or vitiated blood, though less so in the last case. In this case twenty-four teeth are erupted, and all full grown except the canines, which are nearly grown apices, very open and funnel shaped.

### Case 5—18 years old.

In this case all the teeth except the dens sapientia are erupted and full grown, except the second molars, which are open and funnel shaped and very thin edges.

The sockets are fully formed, but the fangs do not rest on the bottom.

In this case these last named teeth must have been erupted at least six or seven years, and fangs still widely open.

### Case 6—18 years old.

Evidently this case is older than stated. All the teeth are fully developed and perfected, except the left inferior wisdom, the crown of which is just above the bone. The fang not fully grown, and open and funnel shaped, would have been closed by the time it was fully erupted.

In this case the jaws are well developed and teeth sound, indicating soundness of constitution. All the others were more delicate in osseous structure.

From the above specifications how is it possible in the living subject to determine at what precise time the fangs are perfectly formed and calcified at apex. Right here lies an important problem in relation to destroying nerves in young teeth by the introduction of arsenic, and ultimately filling the fangs. How are we to judge of the special condition at certain ages of teeth. Some index to the degree of apicial completion may be had by the character of the mouth, including the teeth and general osseous development.

Suppose arsenic to be introduced into teeth with open and funnel shaped fangs, and to remain there twenty-four or forty-eight hours, what would be the probabilities in such cases; in my judgment, inevitable loss of such teeth, from the fact that the poison must necessarily reach the socket and there work mischief.

Now again, suppose the nerve to be destroyed in any other way, what are the probable chances of filling such fangs successfully with any kind of filling, even knowing the actual state of things; decidedly in the negative, as the apicial openings in such cases are larger than the canal higher up in the fang, which would render all attempts at filling such fangs abortive, even if filled by a plug of wood or anything in the form of a cylinder, that might be successfully introduced and forced down near the apex without actually reaching the socket, the operation must necessarily prove a failure from the fact that the fang has actually no vital connection with the floor of alveolas or socket; which connection being a subsequent act after complete calcification of the fang. I need not explain farther, as the difficulties will be readily recognized by the intelligent Dentist.

The microscope shows clearly that calcification commences at the periphery of the dentine, and travels inwards and downwards toward the apex of fang and until the fangs are fully completed. The cement consists of a very thin layer of uniform thickness throughout the fang, just thick enough to give attachment with the alveolus. After the completion of dentine, then the cement begins to thicken over the end of fang and gradually travels up the same, and when *tout ensemble* we find a gradual tapering of cement in thickness from apex to neck.

What in relation to the *heroic* use of the mallet in such young teeth, even with or without live nerves.

Another nut to crack. A large molar tooth has ten millions of terminal nerve filaments, and future calculations may more than double that number.

[TO BE CONTINUED.]

